# Plant based dietary supplement increases urinary pH

**DOI:** 10.1186/1550-2783-5-20

**Published:** 2008-11-06

**Authors:** John M Berardi, Alan C Logan, A Venket Rao

**Affiliations:** 1 Precision Nutrition Inc, 1665 Gregory Road, St Catharines, ON L2R 6P9, Canada; 2 Integrative Care Centre of Toronto, 3600 Ellesmere Road, Unit 4, Toronto, ON M1C 4Y8, Canada; 3 Department of Nutritional Sciences, University of Toronto, 50 College Street, Toronto, ON M5S 3E2, Canada

## Abstract

**Background:**

Research has demonstrated that the net acid load of the typical Western diet has the potential to influence many aspects of human health, including osteoporosis risk/progression; obesity; cardiovascular disease risk/progression; and overall well-being. As urinary pH provides a reliable surrogate measure for dietary acid load, this study examined whether a plant-based dietary supplement, one marketed to increase alkalinity, impacts urinary pH as advertised.

**Methods:**

Using pH test strips, the urinary pH of 34 healthy men and women (33.9 +/- 1.57 y, 79.3 +/- 3.1 kg) was measured for seven days to establish a baseline urinary pH without supplementation. After this initial baseline period, urinary pH was measured for an additional 14 days while participants ingested the plant-based nutritional supplement. At the end of the investigation, pH values at baseline and during the treatment period were compared to determine the efficacy of the supplement.

**Results:**

Mean urinary pH statistically increased (p = 0.03) with the plant-based dietary supplement. Mean urinary pH was 6.07 +/- 0.04 during the baseline period and increased to 6.21 +/- 0.03 during the first week of treatment and to 6.27 +/- 0.06 during the second week of treatment.

**Conclusion:**

Supplementation with a plant-based dietary product for at least seven days increases urinary pH, potentially increasing the alkalinity of the body.

## Background

The influence of net dietary acid load on human health has been the subject of increased scientific investigation over the last decade. Much of the research has focused on the connection between increased dietary acid load via the Western diet and its impact on osteoporosis. Since the bone matrix contains a relatively abundant alkali reserve in the form of calcium and magnesium cations, it may be called upon to buffer the tightly regulated blood pH in the presence of an overly acidic diet [1]. Foods and beverages which have potential to contribute to the net acid load in the human body are said to have a high, or more positive, potential renal acid load (PRAL). Conversely, foods and beverages which are abundant in potassium, bicarbonate and alkaline minerals are said to have a lower, or more negative, PRAL score [2]. Indeed, research does indicate that frequent consumption of high PRAL acid-forming foods (cheese, meats, processed grains) and infrequent consumption of potassium and bicarbonate-rich, alkaline-forming foods (fruits and vegetables) is associated with increased urinary calcium and magnesium loss and a greater risk of osteoporosis [3-6]. In the field of nutritional anthropology, researchers have estimated that the current dietary PRAL per day has increased some 3-fold vs. the hunter-gather diet of the Paleolithic period [7].

In addition to the growing body of research which shows a strong connection between dietary acid load and bone health, other preliminary investigations are uncovering links between an acid-heavy diet and obesity, cardiovascular disease, as well as overall health and well-being [8-10]. The common thread among these lines of investigation may be the stress hormone cortisol. Researchers have shown that the typical acid-heavy Western diet promotes cortisol production [11], and this elevation in cortisol can be attenuated when the acidic diet is neutralized via bicarbonate supplementation or a low acid-diet [12,13]. Since elevated cortisol has been associated with obesity, cardiovascular disease and mental health [14-16], this may be at least one mechanism whereby an alkaline diet, or neutralization of an acidic diet, might promote lean body mass and promote a positive mental outlook.

The emerging research on dietary and net acid load has specific implications for sports nutrition investigators and clinicians for several reasons. First, it is known that intense exercise induces a state of metabolic acidosis, and the buffering required in this process leads to disturbed mineral homeostasis and increased urinary calcium excretion [17]. Second, the higher protein intakes of body builders and other athletes is known to increase urine acidity and calcium loss [18]. Finally, the pre-exercise state of systemic pH and blood buffering capacity through pre-exercise dietary components may significantly influence recovery kinetics and endurance capacity during multiple exercise bouts [19].

Certain dietary supplements, particularly the so-called "green food" category of powdered fruit, vegetable and herbal extracts, are often purported to have an alkaline influence in the human body. One such commercially available green food supplement, greens+ (Genuine Health, Toronto, Canada), has been the subject of at least one controlled trial in a university setting (see table 1 for ingredients list). The product was shown to improve energy in adults, with trends toward improving well-being and mental health vs. placebo [20]. While there is general consensus that diet can undoubtedly impact acid-base balance, and that an individual's net acid load can be specifically modified by dietary interventions [21], the area of dietary supplements and acid-base balance is an unknown. With few exceptions such as potassium and bicarbonate supplements, it is unclear if most dietary supplements can influence urinary pH at all. Since urinary pH has recently been shown to be a reliable indicator of dietary acid-base load via fruit, vegetable and meat intakes [22], we decided to undertake a preliminary investigation of greens+ to determine if it the food-based supplement greens+ might influence first morning urinary pH. (see table 2 for PRAL score of greens+).

**Table 1:** Ingredients in an 8.5 g serving of greens+

**Ingredients per 8.5 g serving of supplement**		
Phosphatide complex (26% phosphatidyl choline from 97% oil-free lecithin)	2,171	mg

Organic barley, alfalfa and wheat grass, and red beet powders	1,543	mg

Spirulina	1,450	mg

Apple fibre powder	1,033	mg

Japanese chlorella (cracked cell)	383	mg

Organic soy sprout powder	383	mg

Organic whole brown rice powder	383	mg

Stevia leaf powder	225	mg

Eight non-dairy bacterial cultures containing Lactobacilli and bifidobacteria (2.5 billion per serving) in a special base of fructo-oligosaccharides (FOS)	200	mg

Royal jelly (5% 10-HDA)	150	mg

Bee pollen powder	150	mg

Licorice root extract standardized to 10% glycyrrhizin (5:1 = 580 mg)	116	mg

Acerola berry extract standardized to 18% Vitamin C	115	mg

Siberian ginseng extract standardized to 0.8% eleutherosides (28:1 = 1,680 mg)	60	mg

Milk thistle extract standardized to 80% silymarin (15:1 = 900 mg)	60	mg

Organic Atlantic dulse powder	33	mg

Ginkgo biloba extract standardized to 24% ginkgo flavonglycosides and 6% terpene lactones (50:1 = 1,000 mg)	20	mg

Japanese green tea extract standardized to 90% polyphenols (20:1 = 300 mg)	15	mg

European bilberry extract standardized to 25% anthocyanidins (100:1 = 1,000 mg)	10	mg

Full spectrum grape extract standardized to 95% proanthocyanidins and 500 ppm Resveratrol (500:1 = 2,500 mg)	5	mg

**Table 2:** Mean PRAL scores (mEq) per 8.5 g serving of greens+; averaged over 10 samples.

Sample #1	-2.3 mEq
Sample #2	-2.5 mEq

Sample #3	-1.7 mEq

Sample #4	-2.5 mEq

Sample #5	-2.1 mEq

Sample #6	-2.4 mEq

Sample #7	-1.7 mEq

Sample #8	-2.4 mEq

Sample #9	-1.8 mEq

Sample #10	-1.5 mEq

Mean +/- SEM	-2.1 +/- 0.03 mEq

## Brief report

After giving informed consent, 34 adults (16 women and 18 men; 33.9 +/- 1.57 y, 79.3 +/- 3.1 kg) participated in this investigation. These participants were not taking any prescription or over-the-counter medications, nor were they taking any nutritional supplements. Further, each reported having no diagnosed health issues. Upon enrollment, participants were asked to refrain from making any significant changes to their dietary intake and/or exercise habits. In addition, they were asked to avoid introducing dietary supplements during the course of the 21 day investigation.

Each participant was also provided urinary dipsticks (AimStrip, Germaine Laboratories, San Antonio TX) which were graded to measure pH from 5.0 to 8.5. Participants were instructed to collect the first morning urinary void in a clean and dry glass container, and measure its pH for seven consecutive mornings in the absence of nutritional supplementation. These measures were collected by dipping the pH strip into the container of urine for one second and, after 60 seconds, comparing the resultant color to a predefined color chart provided the participants. Once a color match was achieved, the corresponding pH value was recorded. This initial seven day period not only allowed us to determine the baseline pH of this group, it also allowed each participant to act as their own control.

On the eighth day, and continuing through an additional two weeks, participants initiated supplementation with two divided servings per day. It was requested that the supplementation be consumed once in the am and once in the pm, each time mixing one tablespoon of product (8.5 g) with 8 oz of water. As above, first morning urinary pH was measured during the 14-day treatment period. Note: the supplements were provided in unlabelled containers so that the participants were unaware of the particular brand of product being studied or its intended use.

Once all 21 days of data were collected, data were analyzed using repeated measures ANOVA (SPSS, Version 10, Chicago, IL). For significant main effects, LSD pairwise comparisons were conducted. All values are reported as mean +/- SEM and statistical significance was set at p ≤ 0.05.

**Figure 1:**
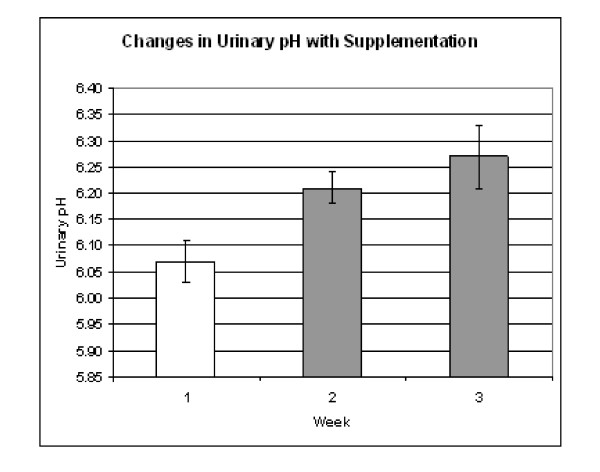
**Mean pH values +/- SEM before and during supplementation.** pH during weeks 2 and 3 were significantly greater than during the baseline period (p = 0.031 and 0.032 respectively).

The mean urinary pH during the one week baseline period was 6.07 +/- 0.04 compared to 6.21 +/- 0.03 (p = 0.031 vs. baseline) during the first week and 6.27 +/- 0.06 during the second week (p = 0.032 vs. baseline). The data are presented in graph form in figure 1.

Of the 34 participants, 21 of them (62%) observed an increase in pH with supplementation while 10 of them (29%) observed a decrease and 3 of them (3%) remained the same. Interestingly, the participants that saw the increase in pH began with the lowest baseline pH values, 5.97 +/- 0.02, which then increased to 6.37 +/- 0.01 with supplementation. And the participants that saw the decrease in pH began with substantially higher baseline pH values, 6.39 +/- 0.02, which then decreased to 6.12 +/- 0.01 with supplementation. The individual per subject data is presented in table 3.

**Table 3:** Mean pH values before supplementation and during two-week supplementation period by subject.

Participants with pH Increases (n = 21)			Participants with pH Decreases (n = 10)			Participants with no pH Changes (n = 3)		
Subject	pH at baseline	pH with supplementation	Subject	pH at baseline	pH with supplementation	Subject	pH at baseline	pH with supplementation

#1	6.0	7.00	#3	7.00	6.00	#4	5.00	5.00

#2	6.29	6.39	#5	5.79	5.73	#13	6.07	6.07

#8	5.90	6.07	#6	6.14	6.11	#17	6.00	6.00

#9	6.29	6.64	#7	7.07	6.68			

#10	6.36	6.50	#15	6.43	6.14			

#11	6.36	6.75	#20	6.43	6.04			

#12	5.50	6.04	#22	6.50	6.25			

#14	6.29	6.46	#24	6.14	6.00			

#16	5.86	6.32	#28	6.29	6.21			

#18	6.07	6.11	#34	6.07	6.04			

#19	6.21	6.61						

#21	5.67	5.83						

#23	6.14	6.50						

#25	5.71	6.13						

#26	5.79	6.43						

#27	6.00	6.24						

#29	5.91	6.00						

#30	6.29	6.50						

#31	6.00	7.00						

#32	5.00	6.00						

#33	5.79	6.26						

Mean +/- SEM	5.97 +/- 0.02	6.37 +/- 0.01	Mean +/- SEM	6.39 +/- 0.02	6.04 +/- 0.01	Mean +/- SEM	5.69 +/- 0.02	5.69 +/- 0.02

On the whole, these data indicate that in this population, mean urinary pH tends to shift from more acidic to more basic with supplementation. However, it also seems that those with the most acidic baseline urinary pH values seem to benefit from supplementation than those with more basic baseline urinary pH values. The reason for this difference is unclear and points out a need for further research in this area.

## Discussion

As the research begins to gather strength, it is becoming increasingly clear that the net acid load of the typical Western diet has the potential to influence many aspects of human health, most notably osteoporosis. Diet-induced, low-grade metabolic acidosis was first described by Japanese urologist Yoshinari Katoh when he reported on animal protein and renal stone formation [23]. When a daily diet with a high net acid load becomes the rule rather than the exception, this chronic, low-grade level of metabolic acidosis may influence bone integrity and other aspects of human health [24]. Diet-induced low grade acidosis may compound some of the negative consequences of metabolic acidosis induced by exercise, not the least of which might be a compromised performance and recovery time during intermittent exercise [19]. Despite considerable expense, great intentions, and impressive marketing by government and professional dietetic groups, there remains a considerable segment of the North American populace who are resistant to the messages concerning the health properties of fruits and vegetables. The majority are still well short of consuming even the minimum five recommended servings of fruits and vegetables, particularly when frozen-fried potatoes are removed from consideration as a "vegetable" serving [25]. This lack of dietary fruits and vegetables most often translates into a net loss of fiber, antioxidants and acid-buffering capacity.

The significant changes to urinary pH noted in our study suggest that plant-based dietary supplements (greens+, in this case) might play a beneficial role in helping to neutralize the net acid load of the Western diet. In particular, the intervention appeared to make the biggest difference in those who had a more acidic pH at baseline. While the introduction of dehydrated, powdered food-based dietary supplements is certainly not a substitute for making every effort to consume a diet rich in fruits and vegetables, it may represent a straight-forward means to at least minimize the effects of a high dietary acid load.

In the end, this was an exploratory study to determine if a powdered plant-based supplement has the potential to influence a key marker of acid-base balance, urinary pH. Obviously no broad conclusions can be drawn, and the findings should be interpreted as simply an impetus for further research in this area. This initial study did not estimate the dietary PRAL of the participants, nor did it consider cortisol, lifestyle habits, stressors, or urinary output of minerals such as calcium. Further, the study did not collect 24-h urine samples to determine 24-h urinary pH. It is also true that having the subjects act as their own control removed randomization as a factor, and this should indeed be acknowledged as a limitation regarding the results.

This was, quite simply, an initial investigation to determine if a plant-based dietary supplement can influence urinary pH. As a result, clearly, there are many unanswered questions regarding dietary supplements and net acid load, and our own group plans to expand on this research with further evaluations of powdered plant-based food supplements with varying ingredient profiles. We hope to determine what, if any, influences they may have on cortisol and additional acid-base health markers.

## Competing interests

Genuine Health Inc, Toronto, Canada provided the nutritional supplements and urinary dipsticks used in this study. Each of the authors involved in this investigation occupies a position on the research board of Genuine Health, the manufacturer of the plant-based nutritional supplement used in this investigation. Within the past five years, AL has been a compensated independent consultant for genuine Health. However, he, nor any author holds stock or shares in the company, nor do they receive compensation that is tied directly to product sales. The product used is not patented or under active patent application. There are no other competing interests to report among any of the authors.

## Authors' contributions

JMB participated at the lead author and was responsible for the study design, screening and recruitment, data collection, data analysis and interpretation, and for the final draft of the manuscript. ACL assisted in the study design, the data analysis and interpretation, and in the draft of the final manuscript. AVR also assisted in the study design, the data analysis and interpretation, and in the final draft of the manuscript.
